# Interaction and conflict between outreach workers and research officers in implementing collaborative action research in the field of harm reduction: a qualitative study

**DOI:** 10.1186/s12954-021-00551-y

**Published:** 2021-10-09

**Authors:** Nicolas Khatmi, David Michels, Daniela Rojas Castro, Perrine Roux

**Affiliations:** 1 Aix Marseille Univ, INSERM, IRD, SESSTIM, Sciences Économiques & Sociales de la Santé & Traitement de l’Information Médicale, Marseille, France; 2 AIDES, 14 Rue Scandicci, 93508 Pantin, France; 3 Laboratoire de recherche communautaire, Coalition PLUS, Pantin, France

**Keywords:** Hepatitis C, Drug injection, Collaborative action research, Focus group, Outreach workers

## Abstract

**Background:**

The effectiveness of collaborative approaches in health interventions is underlined in the literature. Given the serious challenges to adequately managing the HCV epidemic in people who inject drugs (PWID), and the need to improve existing harm reduction (HR) interventions in this population, it seems important to investigate how collaboration between stakeholders is ensured in action research interventions. The present study aimed to explore interactions between outreach workers and research officers collaborating in the implementation of an action research project for PWID entitled OUTSIDER.

**Methods:**

Using three focus groups, we studied the views of 24 outreach workers involved in the implementation and evaluation of a harm reduction educational intervention to help PWID inject more safely in off-site settings.

**Results:**

The analysis of participants’ discourses highlighted the mixed perceptions they had about OUTSIDER. Several limitations to collaboration emerged. Epistemological (theoretical vs. practical knowledge), methodological (science vs. intervention), axiological (standardised vs. adapted approach), and material (mobilised vs. available resources) issues all placed a burden on the outreach worker–research officer relationship. Outreach workers’ acceptance of the project’s intervention dimension but rejection of its scientific dimension highlights a lack of contractualisation between the stakeholders involved, and a more general problematisation of the role of outreach workers in implementing action research in HR. How collaboration was perceived and practised by outreach workers participating in OUTSIDER can be considered a reflection of the current challenges to implementing action research in HR.

**Conclusion:**

This study of the interaction between the research and implementation dimensions of an action research project explored the tensions between different intervention stakeholders that must work together. Equitable participation and integration of the expertise, practices, and knowledge of all stakeholders involved is essential for successful action research. Given current HCV epidemiological challenges, new forms of cooperation are needed when developing healthcare services and when strengthening collaborative approaches.

## Introduction

### Collaborative action research in the field of harm reduction

In the field of health, collaborative action research—where researchers and healthcare practitioners collaborate together—is recognised as a key element in improving the effectiveness of innovative interventions [1–3]. This collaboration must ensure that the expertise, practices, and knowledge of all the stakeholders involved are taken into account. Despite this recognition, the literature highlights several barriers to the implementation of this kind of research, including a lack of funding [4] and researcher-specific issues such as unrealistic expectations [5], negative attitudes [6, 7], and differences in stakeholder statuses [8]. These barriers prevent the integration of healthcare practitioners’ expertise when designing action research interventions and hamper equitable collaboration during their implementation.

In the field of drugs and especially injecting drug use, the long-standing collaboration between researchers and healthcare practitioners—in particular outreach workers^1^ in harm reduction (HR) centres—is vitally important. Outreach work ensures that health interventions are adequately implemented on the ground and often act as an entry point for people who use drugs to access a range of health services. It is a key component of all HIV/HCV prevention and HR programmes. Face-to-face contact between outreach workers and PWID is associated with fewer risky behaviours in terms of HIV/HCV infection and increased HIV/HCV screening [9, 10]. However, the continued high prevalence of HCV in PWID [11, 12] highlights that risky injection practices are still very widespread in this population. It also underlines the difficulties in designing and implementing innovative and effective prevention and harm reduction interventions for this population. In order to improve existing HR interventions in the field of injecting drugs, it seems important to investigate how collaboration between researchers and outreach workers is ensured in collaborative action research. The present study aimed to answer this question, by studying the perspectives of outreach workers participating in the ongoing OUTSIDER action research project. Specifically, we aimed to explore how these stakeholders interacted and collaborated with the study’s research officers in the implementation of this project.

### The OUTSIDER action research project

OUTSIDER^2^ (i.e. outreach for safer injecting drugs education research project) is an ongoing collaborative action research project designed by research officers from the association AIDES—which works in the fight against HIV, viral hepatitis, as well as all other sexually transmitted infections and associated pathologies—in collaboration with the French public health research team UMR 1252-SESSTIM^3^.

Initiated in 2016, it aims to both implement and then evaluate a support and safer injection education intervention for PWID named AERLI^4^ in off-site contexts (e.g. squats, public spaces, car parks). Its primary focus is on evaluating unsafe injecting practices, especially in terms of infectious diseases and venous damage.

In order to reach the PWID furthest from care, outreach workers (comprising peer educators, nurses, and trained social workers) from 20 partner HR centres throughout metropolitan France participate in OUTSIDER. Specifically, they either implement the AERLI intervention to PWID enrolled in OUTSIDER or collect data from these PWID which are then used by the joint AIDES-UMR 1252-SESSTIM team to evaluate the intervention. All the outreach workers participating in OUTSIDER are recruited by the managers of the 20 partner HR centres.

### Implementation of AERLI intervention

AERLI consists of providing harm reduction training and education to PWID about HIV and HCV transmission and other injection-related complications. PWID enrolled in OUTSIDER are recruited by outreach workers from the project’s partner HR centres and followed for one year. The intervention is organised as a series (at least one educational session within six months of PWID inclusion in OUTSIDER) of participant-centred face-to-face educational sessions. Each educational session includes

- Direct observation of the individual PWID by two outreach workers trained in AERLI (the PWID self-injects the psychoactive product they usually use);
- Analysis by the trained outreach workers of the PWID’s injecting practices, identification of injection-related risks, and explanation of safer injecting practices;
- An educational exchange on the individual PWID’s injection practices (including answering any questions the PWID might have).

### Evaluation of AERLI intervention

As mentioned above, some outreach workers implement the AERLI intervention to OUTSIDER’s participating PWID, while others administer intervention assessment questionnaires to them at inclusion (M0), 6 months (M6), and 12 months (M12).

### Legal framework of AERLI intervention

All the outreach workers participating in OUTSIDER are trained in the AERLI intervention. The existing legal framework in France stipulates that support for safer injection can only be provided within an experimental framework. Accordingly, participation in OUTSIDER enables the project’s partner HR centres to provide the experimental AERLI intervention to PWID in off-site contexts as part of the range of services they offer.

## Methods

### Study design

To study outreach workers’ perspectives about OUTSIDER and the different representations they had of this project, we used the qualitative method of focus groups. Focus groups provide a forum for exploring the attitudes, perspectives, and impressions of people who share a common experience [13] and reveal different points of view [14].

When organising focus groups, the social context of the activity and the composition of the groups must be considered [15]. Having a mixed statutory context—that is to say having members from different social groups (e.g. profession, gender)—helps stimulate exchanges and reveals the nature of pre-existing relationships, agreements, and tensions, both within a defined social group and between different interacting social groups [15]. This is why we decided to include an OUTSIDER research officer in each of the outreach worker focus groups. Their role was exclusively to stimulate discussion. Neither their perspectives of OUTSIDER nor of the collaboration with outreach workers were studied.

### Data collection

Three focus groups were conducted between October 2017 and March 2018. Each comprised one research officer and eight outreach workers. Outreach workers were recruited from 11 of the 20 partner HR centres collaborating in OUTSIDER. The research officers were recruited from the UMR 1252-SESSTIM research team and the association AIDES. The focus groups took place in a dedicated room located in the premises of both the AIDES association (Paris) and UMR 1252-SESSTIM (Marseille).

A trained facilitator conducted the focus groups. To start the collective discussion, participants were invited to express their opinions and questions about OUTSIDER and the modalities of their participation in this action research project. The groups were organised using a semi-structured guide which covered four themes: (1) outreach workers’ knowledge about OUTSIDER; (2) their perceptions of the project’s value and benefits; (3) their perceptions of the limitations and barriers to OUTSIDER’s implementation and evaluation; and (4) their experience of implementing the AERLI educational intervention to PWID.

After the facilitator clearly described its objective at the outset, each outreach worker had to provide oral consent to participate. The three focus groups were audiotaped and subsequently transcribed. To ensure anonymity, participants’ names were not transcribed and the recordings were destroyed after the transcription.

### Data analysis

We performed a thematic content analysis of the transcribed data [16] which included: (1) coding the data to reduce the polysemy of individual discourses into a set of codes; (2) grouping these different codes into thematic categories in order to identify the relationships between the codes and concepts raised by the participants; (3) analysing exchanges during the focus groups and their variation; and (4) describing and modelling the phenomena identified, by putting the discourses produced and the group dynamics observed into context.

To present the results, we provide quotes from some outreach workers and research officers. Quotes for the former are labelled by the HR centre they belonged to. For example, C1 indicates HR centre 1. Research officers’ quotes are labelled as RO. The facilitator’s prompts are labelled as F and are presented in capital letters here.

## Results

### Perceived benefits of implementing OUTSIDER

Participating outreach workers were very positive about the off-site AERLI educational intervention. Indeed, all declared that their primary interest in participating in OUTSIDER was to receive training to implement AERLI and to improve their working practices and technical knowledge, in order to allow them to “*better help people in difficulty with their injection practices*” (C4).

It initially seemed to me to be a good idea to participate in OUTSIDER, because people can have infected injection sites, and people who don’t inject themselves need help injecting because they don’t know how to (C7).

They also felt that AERLI helped to improve their relationship with PWID. Through implementing the intervention in an off-site context, they wanted to “*maintain the link with the users*” and to “*strengthen the relationship of trust*” (C1), in particular with the most vulnerable users who did not frequent HR structures.

- F: ARE YOU UNDER THE IMPRESSION THAT IT’S MORE DIFFICULT TO TALK ABOUT INJECTION WITH THE PEOPLE FURTHEST FROM THE HEALTHCARE SYSTEM?
- C1: It depends on the relationship you have with them. That’s the most important thing. On the temporality you propose to them. Proposing a project that lasts a year could be difficult … that could be relatively complicated when you live from day to day and… I don’t know.

As mentioned above, the existing legal framework in France allows support for injection only in an experimental framework such as OUTSIDER. In reality, some of the partner HR structures had already implemented AERLI before OUTSIDER was developed. For these centres, participation made it possible to regularise this practice.

- F: WHY ARE YOU PARTICIPATING IN THE OUTSIDER PROJECT?
- C1: Actually, it’s so that we can legally continue doing it [provide support for injecting drug users]. And do it a little more. Because, that way we also avoid, so as not to endanger the structure, but that’s what we want.
- C6: I agree with him on this. From what I’ve heard, it’s that for us, the possibility of providing support for injection isn’t yet in place […] and for everyone, their interest in OUTSIDER is dependent on the continuity of the AERLI interventions, and that it seemed completely logical [for them] to participate.

### *Perceived barriers to implementing* OUTSIDER

First, the outreach workers’ discourses indicated that not all of them had fully evaluated what participating in the AERLI training would involve in terms of the larger OUTSIDER project. Indeed, most had little or no idea about what OUTSIDER really is: *“Why do research on something we’re already doing?”* (C4).

- C3: I don't have a lot of… I admit my shortcomings; I don’t have a lot of information [about OUTSIDER]. I'm sure it was sent to me [the information brochure by email]. I’m sure it’s my fault, eh, but I didn't look for it.
- C1: No, I didn’t inquire about the project. I’ll be straight up. I wasn’t a good student. I didn’t have time to read the information on OUTSIDER. But, then, afterwards I had discussions [about it].

Because of their lack of detailed knowledge about OUTSIDER, the study’s research questions and objectives, its scientific dimension, and related protocol practices (e.g. PWID recruitment and retention; completion of questionnaires to evaluate the intervention’s implementation in an off-site context) were all a source of controversy for the outreach workers and led to their reluctance to actively participate in it. Some felt that the *standardised* scientific approach used in the proposed evaluation study went against their *flexible* and *adapted* on-the-ground practices. For example, they felt that OUTSIDER’s inclusion criteria for PWID were too *strict*, that the conditions for evaluating the AERLI intervention were *tedious*, and that the programme for the off-site AERLI educational sessions was *too formal* and *standardised*. Scientific practices were perceived by the participants as being at odds with intervention practices, the latter being described as more *flexible*, at the *service of PWID* and adapted to their [PWID’] *“living conditions” (C1):*

- F: WHAT ARE THE DIFFICULTIES YOU PERCEIVE FOR THE IMPLEMENTATION OF THE OUTSIDER PROJECT?
- C4: It’s getting them [the users] into the programme and being able to keep them there […]. For one thing, for an AERLI session, you’re on site, and you’ve got someone with his arms ruined and he tells you that he doesn’t know what he can do anymore … so, already that means that you’re going to try to get closer to him and then try to talk to him little by little about AERLI […]. Well, doing AERLI in squats or in people’s homes is already a bit of a battle, and when you get to do it, you’re super happy because you’ve just taken one step forward, and you’re going to be able to help them, ‘educate’ them to do better ... So, if you have to *also* get them into a [scientific] programme ... that’s what I find a little ...

For some participants, the scientific dimension of OUTSIDER hindered their practices and weakened their relationships with PWID. Furthermore, some felt that action research favours the production of scientific knowledge over intervention practices. Criticism of OUTSIDER was based on their knowledge of PWID practices and profiles. For example, they considered that the main scientific objective of OUTSIDER—which is to reach PWID most distant from care (i.e. off-site)—was unsuitable, as the project’s protocol excluded PWID who wished to inject in high-risk body sites, including the neck and groin; in other words, it excluded the very people who tend to be most distant from care. The following quotes illustrate their opposition to the protocol used in OUTSIDER:

- C1: You realise that sometimes the speeches [With regard to the ‘good practices’ of safe injection recommended in OUTSIDER’s protocol] just don’t hold water… You’re in a fantasy world of what is good… […] it’s a delicate issue, because for example, sometimes we give advice like “change the injection site, look for a vein, this one looks good...”. He looks for the vein, he doesn’t find it, he butchers himself, he gets blood everywhere... and you’re right there in front of him. And you say to him “well, do it again like you usually do” because you don’t want him to hurt himself, and in fact you already know that his usual way works fine. [...] you realise that everything that’s understood in the [harm reduction] center or on the mobile unit [With regard to the ‘good practices’ of safe injection recommended in OUTSIDER’s protocol] isn’t necessarily adapted to the street or in spaces that are a bit particular.
- C3: What you’d like is to be able to really see their practices and then be able to work on them. But… that’s it, like, based on that [users’ real-world injection practices]... If you start by demonising what they’re doing, it’s not going to encourage them to come back again and share [their practices and knowledge].

Finally, some outreach workers felt that their participation in OUTSIDER could lead to a situation of conflict, in the sense that intervention practices (i.e. the action dimension) and scientific practices (i.e. the research dimension) were set against each other. The following excerpt presents an exchange between outreach workers from the HR C4 centre and a research officer. It illustrates this conflictual dimension and its impact on the relationship between these stakeholders:

- C4: I have a question about people who aren’t included. [...] that means that at some point if these people who aren’t included in the OUTSIDER programme want to have the AERLI, what do we tell them? Do we tell them “No, you can't because you refused to join the OUTSIDER programme” or is it possible all the same? It might be a stupid question, eh, but...
- RO: They won’t be able to join because their results cannot be compared with others who’ve had the AERLI sessions. So, if a person is in the non-participant group, even if they change their mind, they can’t be part of the intervention group. That’s the protocol, eh.
- C4: [...] personally, I couldn't say no, like, say “no, no you said ‘no’ to me two weeks ago, it’s too late”. I mean, that’s impossible!
- RO: No, they’ll still be able to have it, but wouldn’t be part of the project.
- C4: But that doesn't answer the question […] What you said, that wasn’t my question, it was if a person says ‘no’ at M0. So, your answer is: ‘it’s better that she doesn’t have AERLI for a year in terms of the research protocol’. That’s what I understood from your answer [...] because in the document that was sent to us [the project information brochure], we’ve got a panel of people who participate in OUTSIDER and the idea is to see that, we see that there’s a ‘magic’ effect, that there’s fewer infections, they take care of their veins etc. etc... And another panel which doesn’t participate and we see that ‘oh dear, they all get HCV and suddenly it’s a massacre’... I’m putting it in broad strokes, but the idea is that. And we’ve got a person who says “no, I don't want to participate in this thing [the OUTSIDER project], it’s boring, I don't want to come…” and then who says to us in the end “but, actually, I would like to have AERLI, come to the squat and we’ll do an AERLI session…” My question is: do I say yes or do I say no? Like, I say yes, eh, but in your research protocol I don’t know what’s set out.

### Impact of HR centres’ level of experience on the implementation of OUTSIDER

The valence (i.e. intrinsic attractiveness, whether positive or negative) of the outreach workers’ attitudes towards OUTSIDER differed depending on the HR centre they worked in. Those from centres where off-site activities were more recent had a more positive valence and a more neutral relationship to the project’s scientific dimension: “*Yes, I read the OUTSIDER brochure and all; well, overall, it might be interesting.”* (C2, No injection support experience). Conversely, the most negative valence was observed in workers from HR centres with the longest experience of outreach work and injection support. They also displayed stronger opposition to the scientific dimension:

- While for us at C4, it doesn’t seem very logical to us actually [to participate in OUTSIDER action research project]. We already wonder a lot about why the center is participating in this study, and honestly—and I’m speaking for myself here—I don’t understand why C4 is participating in this OUTSIDER project, because we’re already providing support for injection in our premises, and in outreach [off-site], especially at festivals, and we already wanted to do it, especially during the on-site opening hours on Wednesdays, and in the squats … so, we don’t see the point of participating in this research (C4, six years’ injection support experience).

## Discussion

The analysis of participants’ discourses highlighted the mixed perceptions they had about OUTSIDER. Several limitations to the collaborative dimension of action research emerged in the discourse analysis. Epistemological, methodological, axiological, and material issues all put stress on the outreach worker–research officers’ relationship. These findings reflect those in the literature on collaborative action research and therefore can be considered representative of action research in HR context. We investigate these issues in more detail below.

### Epistemological issues

Our analyses suggest that the criticisms formalised by outreach workers in their discourses arose from a fundamental dichotomy between intervention practices and scientific practices implemented in the project. More specifically, to distinguish these different practices of the project, they used a system of shared knowledge, values, and references. This system was shaped by their relationship to on-the-ground realities, including their knowledge of technical constraints in areas where OUTSIDER was supposed to be implemented, the specificities of observed user practices, and their experience of implementing the AERLI educational intervention in contexts of social deprivation (e.g. squats, in the street). Respect for the reality of users’ practices emerged as one of the primary dimensions of this system.

By contrasting OUTSIDER’s scientific objectives with on-the-ground realities, outreach workers pitted their practical and contextualised knowledge against the theoretical and standardised knowledge that forms the basis of research work in the field of drugs. Yet the whole reasoning behind implementing collaborative action research is to combine these very two dimensions through a “hybridisation of practices” [17]. This same tension between scientific and non-scientific knowledge is also seen in the literature [18–21]. Our finding highlights the importance of discussing the polarisation of knowledge as part of any epistemological problematisation of collaborative action research.

### Methodological issues

It was through an *invocation to reality* that participants evaluated operational methods and knowledge on which scientific practices are based. Accordingly, they mobilised evaluation criteria based on the conditions of use of the knowledge mobilised, such as the compatibility of practices and the tangible usefulness of knowledge. Obstacles to implementing this action research project were associated with a perceived lack of adaptability of scientific practices (i.e. based on conceptual theories) to intervention methods (i.e. based on the on-the-ground realities discussed above). This finding reveals that the two types of practices mobilised to implement OUTSIDER were perceived as incompatible and that the resulting tension stemmed from the “twin ambition” of action research which seeks to both develop intervention practices and advance scientific knowledge [22]. This perceived incompatibility reflects the fundamental methodological questions that need to be first answered when considering the implementation of action research.

### Axiological issues

Outreach workers in OUTSIDER’s focus groups perceived and evaluated the validity of action research (i.e. methods, objectives, issues to study) based on their own value system. The coexistence of intervention practices and scientific practices placed outreach workers in an “axiological tension” [23] between the values which form the foundation for these two practices. The literature shows the importance of sharing moral [24], political [4], ethical [7], and democratic [25] values when collaborating in health research. This conflict of values appears to be a limitation to the participation and involvement of outreach workers in action research in HR.

### Organisational and material issues

It is important to remember that the managers of the collaborating HR centres—not the project’s research officers—recruited the outreach workers for OUTSIDER. The heterogeneity in the latter’s level of knowledge about the project and in their interest in participating in it, reveals a lack of contractualisation between the two types of stakeholders. Action research involves mobilising professional practices which are evaluated for their effectiveness. Without prior harmonisation of aims and objectives between research officers and outreach workers, this evaluative dimension can produce legitimate resistance to change by the latter, especially those with the most experience.

Furthermore, participation in action research implies *ipso facto* additional duties and increased workload for outreach workers, in a setting where professional resources are already limited (i.e. the time allocated to activities; the technical skills mobilised). During the implementation of any action research project, if the research process is not completely understood and validated upstream by all stakeholders, participants may feel that there is not any direct benefit for them and therefore may consider that the investment needed (i.e. the workload) is not worth the effort.

These organisational issues may explain, at least in part, why outreach workers were very critical of OUTSIDER, and displayed power struggles with the project’s research officers. This result underlines the importance of the material dimension of action research projects—in terms of working time, skills, knowledge, etc.—to ensure their collaborative and efficient implementation and, in turn, to ensure the success of public health interventions in HR.

### Institutional issues

The data collected in the present qualitative study reveal the impact of structural factors, specifically legal frameworks, on the implementation and effectiveness of action research in HR. The existing legal framework in France only allows support for safer injection to be provided in experimental contexts (e.g. OUTSIDER). Accordingly, this implies that participating outreach workers in OUTSIDER had to first accept the scientific issues and methods involved if they wished to legally implement their practices on the ground. This subjugation of their objectives to those of institutional research effectively led to institutional hierarchy between research officers and outreach workers.

All the above results put into question the equitable collaborative dimension fundamental to HR action research [5, 26], as they suggest that power is not shared equally, something already highlighted in the literature for other areas of health [8, 27, 28]. Indeed, action research stakeholders who are not research officers more often play consultative rather than collaborative roles [6, 29]. Accordingly, the stance adopted by outreach workers in the present study may also be considered a defensive tool, or tool of resistance, to institutional and scientific domination [30].

In our opinion, the prioritisation of scientific practices and knowledge—imposed by France’s current legal framework regarding support for drug injection—over field-based practices and needs, only serves as an obstacle to effective collaborative action research and therefore to implementing effective interventions.

The present study has several limitations. The impact of each focus group’s facilitator and their status on discourse content was not monitored. Furthermore, socio-demographic characteristics of the participants, which can have a considerable impact on both relational and educational practices, were not taken into account. Finally, the participating outreach workers worked in different regions of France with different distinct sets of issues and experiences. More focus groups need to be conducted to evaluate these factors (i.e. participants’ self-perceived status, their sociodemographic characteristics, the places where they implement interventions (on-site, off-site)) and their potential impact on the discourses produced.

## Conclusion

How collaboration was perceived and practised by outreach workers participating in OUTSIDER can be considered a reflection of the current challenges to implementing action research in HR. Several limitations to collaboration emerged in the discourse analysis. Epistemological (theoretical vs. practical knowledge), methodological (science vs. intervention), axiological (standardised vs. adapted approach), and material (mobilised vs. available resources) issues all placed a burden on the outreach worker–research officer relationship. Outreach workers’ acceptance of the project’s intervention dimension but rejection of its scientific dimension highlights a lack of contractualisation between the stakeholders involved and a more general problematisation of the role of outreach workers in implementing action research in HR.

Equitable participation and integration of the expertise, practices, and knowledge of all stakeholders involved is essential for successful action research. Given current HCV epidemiological challenges, new forms of cooperation are needed when developing healthcare services and when strengthening collaborative approaches.

## Data Availability

The data sets used and/or analysed during the current study are available from the corresponding author on reasonable request.

## Abbreviations

ANRS: National Agency for Research on AIDS and Hepatitis
HR: Harm reduction
PWID: People who inject drugs
HCV: Hepatitis C virus
AERLI: Individually Tailored Support and Education for Safer Injection
OUTSIDER: Outreach safer injecting drugs education research study

## Footnotes

1 Outreach workers: a person who does work designed to help and encourage disadvantaged members of the community.

2 OUTSIDER is funded and supported by the National Agency for Research on AIDS and Hepatitis (ANRS).

3 Neither the association AIDES nor UMR 1252 SESSTIM has any conflict of interest in the work reported here.

4 Accompagnement et Education aux Risques Liés à l’Injection.
